# The effect of primary care on potentially avoidable hospitalizations in France: a cross-sectional study

**DOI:** 10.1186/s12913-020-05132-6

**Published:** 2020-03-31

**Authors:** Gregoire Mercier, Vera Georgescu, Elodie Plancque, Claire Duflos, Annick Le Pape, Catherine Quantin

**Affiliations:** 1 Health Services Research Unit, DIM, CHU de Montpellier, Montpellier, France; 2 UMR CNRS CEPEL, Montpellier, France; 3 DIM, Hopital La Colombiere, 39 avenue Charles Flahault, 34295 Montpellier, France; 4 Agence Regionale de Sante Occitanie, 1025 Rue Henri Becquerel, 34067 Montpellier, France; 5 CHU de Dijon, 2 Boulevard du Maréchal de Lattre de Tassigny, 21000 Dijon, France

**Keywords:** Potentially avoidable hospitalizations, Primary care, Spatial heterogeneity, France

## Abstract

**Background:**

Potentially avoidable hospitalizations are an indirect measure of access to primary care. However, the role and quality of primary care might vary by geographical location. The main objective was to assess the impact of primary care on geographic variations of potentially avoidable hospitalizations in Occitanie, France.

**Methods:**

We conducted a retrospective analysis of claims and socio-economic data for the French Occitanie region in 2014. In order to account for spatial heterogeneity, the region was split into two zones based on socio-economic traits: median pre-tax income and unemployment rate. Age- and sex-adjusted hospital discharge potentially avoidable hospitalization rates were calculated at the ZIP-code level. Demographic, socio-economic, and epidemiological determinants were retrieved, as well as data on supply of, access to and utilization of primary care.

**Results:**

72% of PAH are attributable to two chronic conditions: chronic obstructive pulmonary disease and heart failure. In Zone 1, the potentially avoidable hospitalization rate was positively associated with premature mortality and with the number of specialist encounters by patients. It was negatively associated with the density of nurses. In Zone 2, the potentially avoidable hospitalization rate was positively associated with premature mortality, with access to general practitioners, and with the number of nurse encounters by patients. It was negatively associated with the proportion of the population having at least one general practitioner encounter and with the density of nurses.

**Conclusions:**

This study suggests that the role of primary care in potentially avoidable hospitalizations might be geography dependent.

## Background

Potentially avoidable hospitalizations (PAH, also referred to as admissions for ambulatory care sensitive conditions) are used as indicators of access to primary care [1, 2]. PAH are associated with a substantial increase in costs and threaten patients’ quality of life [3]. They should be avoidable, if patients are treated in a timely and effective way in the primary care setting. Therefore, PAH rates are routinely measured to monitor the quality of primary care.

PAH rates show substantial between- and within-country variations [4]. Some of the explanatory factors are non-modifiable: age and ethnicity [5]. Others might be modifiable outside the health system sector, such as income and education level [6–9]. Inside the health system sector, the factors mostly pertain to the primary care sector. They include prevention, transitional care, patients’ involvement, continuity of care, and access to and utilization of primary care [10–14]. Published evidence suggests a protective role of General Practitioners’ (GP) density and of utilization of GP services [15]. A review of patients with type 2 diabetes showed mixed results regarding the impact of the density of GPs [16] and a positive association between the number of GP visits and the number of PAHs.

Actual utilization of primary care (e.g., number of contacts) is regarded as more relevant than access to primary care, since it is derived from individual-level data, provided that patients’ health status is adequately adjusted for [16]. Additionally, the role of primary care utilization is of particular interest from the perspective of value-based payment. Because bundled payments may include multiple providers in the primary and secondary care settings, they require a thorough understanding of the link between primary care utilization and PAH [17].

However, the evidence about the impact of primary care utilization on PAH is mixed. A recent review mentioned 15 papers that have investigated the role of primary care utilization defined by the number of GP visits [15]. Among these, four suggested a significant and positive association (i.e., more GP visits associated with higher PAH rates), eight suggested a significant and negative association and three were inconclusive. A U-shaped relationship between the number of GP visits and PAH rates was reported in a study performed on a remote Australian population [18]. These apparently conflicting results might be explained by differences in study populations, in health system traits, and in adjustment based on needs [15, 16]. On the one hand, higher number of GP visits may prevent PAH. On the other hand, greater number of GP visits might indicate sicker patients who are likely to require more hospital care, hence the need of good adjustment variables indicating patients’ health status.

In France, considerable geographic variation in PAH has been found. Beyond epidemiological and socio-demographic factors [19, 20], geographic disparities in PAH might be explained by the density of primary care nurses [12] and by GP utilization in the surrounding regions [21]. The latter is explained by the fact that some patients living in a particular administrative area leave that area for GP visits and hospital admission. The density of primary care physicians and nurses is characterized by wide disparities [22]. As regards health care financing in France, pay-for-performance and pay-for-coordination have been implemented and policy makers are considering the implementation of bundled payment schemes for chronic conditions [17]. Furthermore, most of the policy interventions regarding primary care are undertaken at the regional or local level in France. Hence, taking spatial heterogeneity into account in the estimation of the role of primary care on PAH is of particular interest in France due to the presence of geographic disparities in the distribution of physicians and nurses.

Another critical issue when assessing the impact of primary care is the methodological approach implemented. Ecologic analyses (i.e., PAH rates are modeled at a geographic, not individual level) are frequently performed. They are interesting because they provide average estimates and because they take into account factors that cannot be defined at the patient level, such as the density of primary care physicians [15].

However, ecological analyses are prone to spatial autocorrelation and heterogeneity, and thus should account for interactions between geographic units and be checked for the implicit assumption of homogeneity [23]. Indeed, ordinary least square models, which are frequently used in ecological analyses, suppose that the statistical units of observation are independent. This is rarely the case between contiguous administrative areas which do not necessarily correspond to living areas. Spatial models take into account the spatial correlation between units of observation by introducing the information about nearby observations using a neighborhood matrix.

The aim of this paper was to assess the impact of primary care on PAH geographic variations, considering spatial heterogeneity.

## Methods

### Study design

We performed a retrospective analysis of claims and socio-economic data from the French Occitanie region in 2014.

### Data sources and definitions

We used the French national hospital discharge database (*Programme de Médicalisation des Systèmes d’Information* [PMSI]) that includes data from all public and private hospitals. Data were obtained at two administrative geographic levels: ZIP code (*n* = 612) for the hospital discharge data, and city (*n* = 4607) for the other variables. However, all variables were aggregated at the ZIP code level for the modeling.

Age- and sex-adjusted hospital PAH rates were calculated using the definition developed by the Agency for Healthcare Research & Quality (AHRQ) for chronic conditions (see Table, Supplemental Digital Content 1, which shows the definition of PAH). We chose this definition because it has been adapted to the ICD10 by Thygesen and colleagues [4] and because it is widely used in European countries.

Data on determinants were obtained from the Ministry of Health [24], the National Institute for Statistics and Economic Studies [25], the Regional Health Observatory [26], the Health Insurance Fund ambulatory care claims database (Systeme National d’Information Inter-Regimes de l’Assurance Maladie [SNIIRAM]), and the Regional Health Agency of Occitanie. Demographic and socio-economic data were from the last available French census data, i.e., 2012. The Couverture Maladie Universelle Complémentaire (CMU-C) is a health insurance program for low-income people. The age and sex-adjusted all-cause and premature mortality rates, computed from the number of persons who died before the age of 65, and the proportion of the population exempt from copayments because of chronic disease (severe heart failure, diabetes or severe chronic respiratory insufficiency) were used as proxies for population health status. Primary care supply was defined using densities of general practitioners and of ambulatory care nurses; by the travel time from the center of the ZIP code to the closest emergency department, acute care hospital and medical group practice; and by the presence of at least one ambulatory care center and nursing home service within the ZIP code. Access was estimated using the local potential accessibility measure (Accessibilite Potentielle Localisee [APL]), which is an aggregated measure taking into account densities, travel times and needs. Primary care utilization was defined based on ambulatory care claims data for GPs, nurses, physiotherapists, dentists and specialist physicians. This is a larger definition of primary care, which usually includes only GPs and nurses. Encounters with dentists are used by regional health authorities as a marker of peoples’ behavior in regard to healthcare. Regarding specialist physicians, four specialties have been included because these specialists are directly involved in the ambulatory care management of chronic diseases linked to PAH (Table 1), namely cardiologists (congestive heart failure and angina), pulmonologists (asthma and COPD), endocrinologists and nephrologists (Diabetes Mellitus).

**Table 1:** Frequency of Potentially Avoidable Hospitalizations Clinical Categories in Occitanie, 2014

Clinical category	N	Percent
Congestive heart failure	12,504	47.2
Chronic obstructive pulmonary disease	6652	25.1
Angina without procedure	3781	14.3
Dehydration in elderly people	2119	8
Asthma in adults	1050	4
Diabetes short-term complications	406	1.5
TOTAL	25,567	100

For each of the aforementioned groups, we defined the proportion of the population with at least one encounter during the year, the annual average number of encounters per inhabitants, and the annual average number of encounters per people with at least one encounter. Each variable was obtained after indirect age and sex standardization using the region Occitanie as the reference population. Hence, for each index, a value above 1 means a higher utilization rate than the regional average. All variables are available online (see Table Supplemental digital content 2, which presents all of the variables considered in the statistical analysis).

### Statistical analyses

The outcome was the age and sex-standardized PAH rate by 1000 persons, calculated for each ZIP code. Adjustment variables were preselected using principal component analysis. The objectives were not to include highly correlated factors and to select the most discriminating factors with regard to the PAH rate (see Additional file 2 for a list of all variables explored by PCA and their details). First, an ordinary least square (OLS) regression model was estimated for the Occitanie region. The unit of observation was the ZIP code, the dependent variable was PAH rate and the independent variables were all the variables preselected by PCA (full model). A second model was fitted (reduced model), in which we kept all independent variables whose *p*-values were less than 0.1 in the full model. Since exploratory spatial analysis confirmed spatial disparities in the rate of PAH and Moran’s I test showed residual positive spatial autocorrelation in the OLS model (meaning that nearby ZIP codes had similar PAH rates), a spatial lag model was estimated using the contiguity matrix of the ZIP codes as a spatial autocorrelation matrix. The spatial lag model introduces a lag on the outcome variable (PAH rate). There are several theoretical motivations for the observed dependence between nearby observations, for instance technological interdependence between regions, behavioral modifications depending on neighbors, physical and human capital externalities [27].

The observed variation in the dependent variable may also result from unobserved or latent variables, related to culture, infrastructure and other factors for which data is missing. In this case the spatial lag model will account for omitted variables that influence PAH rate.

The selection among types of spatial models (spatial error model, spatial lag model, spatial Durbin model) was performed according to the sequential test procedure recommended by Anselin and Florax, 1995 [28], based on the principle of parsimony: the model with the fewer parameters that performed well according to our statistical indicators was selected. We used two statistical indicators to test if our model was performing correctly: Moran’s I test for residual autocorrelation (used on the residuals of the model, a *p*-value below 0.05 indicates remaining spatial autocorrelation in the model), and the Breusch-Pagan test for heteroscedasticity (used on the residuals of the model, a p-value below 0.05 indicates remaining heteroscedasticity in the model).

**Fig. 1:**
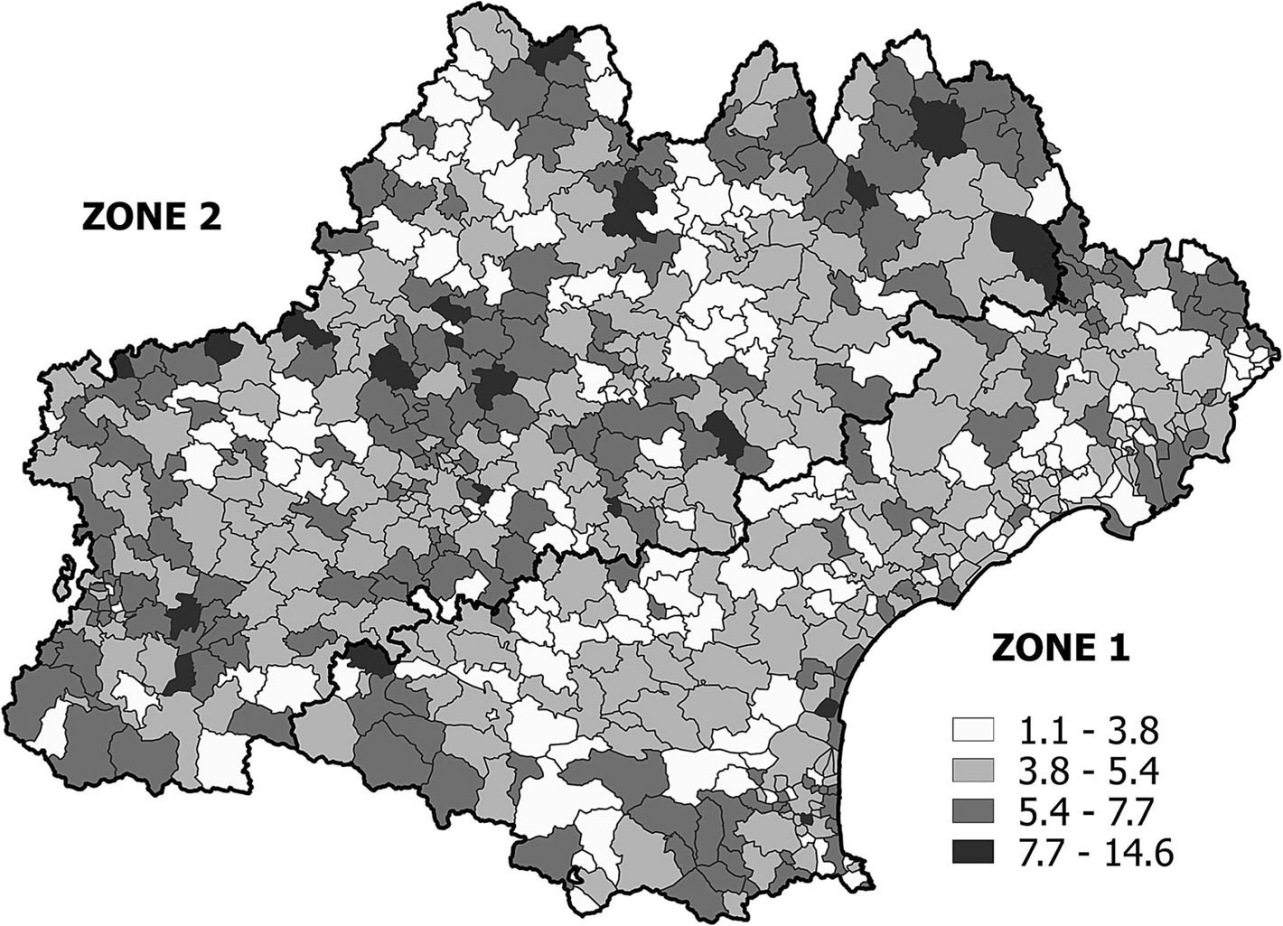
Standardized PAH rates at the ZIP code level. Legend: Figure 1 was generated by authors using Geoda and Arcgis

According to the Breusch-Pagan test, significant heteroscedasticity remained in the spatial lag model (i.e., random regression error did not have a constant variance over all observations), which can indicate regional differences in the relationships modeled (i.e., spatial heterogeneity). Since the Occitanie region exhibits strong socio-economic disparities, we split the region into two homogeneous zones based on the median pretax income and unemployment rate (Fig. 1). Zone 1 is the economically deprived area, characterized by a higher unemployment rate (11.1%, compared to 7.7% in Zone 2) and a lower median income per year (18,247 euros vs 19,102 euros), more single parent families (9.4% vs 7.7%) and a higher number of CMU-c recipients (10.4% vs 6.1%). A full description of the two zones is given in Table 2.

**Table 2:** Descriptive statistics

	Occitanie		Zone 1		Zone 2	
	Mean (SD)	Correlation with PAH rate	Mean (SD)	Correlation with PAH rate	Mean (SD)	Correlation with PAH rate
PAH rate (/1000/year)	4.85 (1.59)		4.59 (1.40)		5.06 (1.71)	
**Socio-economic and demographic data**						
Income (Euros)	18,712 (2194)	− 0.034	18,247 (2087)	− 0.202	19,102 (2208)	0.023
Single-parent families (%)	8.4 (2.1)	0.016	9.4 (1.9)	0.128	7.7 (1.8)	0.056
Baccalaureate education level (%)	40.6 (7.7)	−0.122	40.8 (7.3)	−0.268	40.4 (7.9)	−0.027
Workers (%)	18.4 (5.5)	0.104	18.2 (4.1)	0.151	19.5 (5.6)	0.08
Unemployment (%)	9.2 (2.8)	−0.007	11.1 (2.6)	0.277	7.7 (2.0)	−0.04
Isolated rural areas (%)	25.7 (39.3)	−0.04	18.0 (34.8)	−0.051	32.2 (41.9)	−0.079
CMU-c recipients (%)	8.1 (4.7)	0.062	10.4 (5.1)	0.284	6.1 (3.4)	0.022
**Health status**						
Premature mortality (/100,000/year)	185 (55)	0.143	198 (56)	0.293	174 (51)	0.102
Exemption for chronic disease (%)	9.5 (2.3)	0.048	9.3 (2.1)	0.221	9.6 (2.5)	−0.067
**Primary care supply**						
Density of GPs (/1000)	10.4 (5.8)	−0.01	11 (5.7)	0.027	9.9 (5.9)	−0.012
Density of nurses (/1000)	23.6 (11.2)	−0.114	27.1 (10.5)	−0.019	20.7 (11)	−0.113
Access to GPs	69.7 (24.3)	0.046	73.7 (22.8)	0.027	66.2 (25.1)	0.098
Access to nurses	135.7 (58.8)	−0.076	164.7 (60.9)	−0.01	111.3 (44.1)	−0.008
Access to physiotherapist	76 (43.5)	−0.047	90.7 (46.8)	0.025	64 (36.2)	−0.022
Time to emergency department (min)	17.6 (9.8)	−0.006	15.9 (9.3)	−0.046	19.1 (10)	−0.022
Time to group practice (min)	38.2 (17.3)	−0.006	41.8 (15)	0.091	35.2 (18.4)	−0.014
**Primary care utilization**						
At least 1 GP encounter	1 (0.2)	−0.043	1.04 (0.26)	0.008	0.96 (0.13)	− 0.045
GP encounters (population)	0.96 (0.17)	−0.019	1.01 (0.16)	0.023	0.93 (0.17)	0.011
GP encounters (patients)	0.97 (0.10)	0.041	0.98 (0.1)	0.024	0.96 (0.09)	0.076
At least 1 nurse encounter	1.17 (0.36)	−0.025	1.15 (0.34)	0.018	1.20 (0.37)	−0.069
Nurse encounters (population)	0.91 (0.35)	0.001	1.09 (0.36)	0.068	0.76 (0.25)	0.104
Nurse encounters (patients)	0.84 (0.41)	−0.003	1.03 (0.44)	0.035	0.68 (0.28)	0.114
At least 1 physiotherapist encounter	0.94 (0.24)	−0.055	1.01 (0.23)	−0.028	0.88 (0.22)	−0.002
Physiotherapist encounters (population)	0.90 (0.31)	−0.083	1.05 (0.31)	−0.039	0.77 (0.25)	0.002
Physiotherapist encounters (patients)	0.94 (0.17)	−0.094	1.03 (0.17)	−0.025	0.87 (0.12)	−0.019
At least 1 dentist encounter	0.96 (0.22)	−0.076	1.01 (0.25)	−0.05	0.92 (0.19)	−0.049
Dentist encounters (population)	0.94 (0.21)	−0.094	1.00 (0.21)	−0.079	0.89 (0.20)	−0.044
Dentist encounters (patients)	0.98 (0.08)	−0.05	1.00 (0.08)	−0.046	0.97 (0.07)	−0.001
At least 1 specialist encounter	0.92 (0.21)	−0.01	0.96 (0.18)	0.024	0.88 (0.24)	0.014
Specialist encounters (population)	0.91 (0.30)	0.082	0.92 (0.23)	0.11	0.89 (0.35)	0.082
Specialist encounters (patients)	0.97 (0.18)	0.148	0.95 (0.14)	0.165	0.99 (0.20)	0.122

The same steps were followed for the two zones as for the entire region. First an OLS model was fitted with all preselected variables (full model), followed by a reduced model which kept variables with *p*-value less than 0.1 in the full model and finally a spatial lag model, a spatial error model and a spatial Durbin model were estimated in each of the two zones. After comparison of the three spatial models, the spatial lag models were selected as the final result, following the same parsimony principle as before. These models quantified the specific impact of each factor, excluding the impact of spatial relations between geographic areas (i.e., ZIP codes).

Confidence intervals for parameter estimates were obtained using 5000 MCMC simulations after a burn-in of 500 iterations.

We used SAS, R (package spdep for the estimation of spatial models and confidence intervals obtained by MCMC simulations), Geoda and Arcgis software packages.

## Results

In total, there were 26,512 hospital discharges identified as potentially avoidable in 2014 in the Occitanie region, corresponding to 22,287 patients. These patients had a mean age of 76.4 years (SD: 13.8) and 48% of them were female. The most frequent categories were congestive heart failure and chronic obstructive pulmonary disease (COPD) (Table 1).

At the ZIP code level, the mean age- and sex standardized annual PAH rate was equal to 4.85 per 1000 inhabitants (SD: 1.59) and ranged from 1 to 14.6 cases. Figure 1 presents a map of the spatial variation in PAH rates at the ZIP code level in the Occitanie region.

As expected, Zone 1 had a less favorable socio-economic profile with a higher deprivation index, a higher unemployment rate and a lower median income (Table 2). In addition, Zone 1 had poorer population health, a better health care supply and higher primary care utilization.

In the spatial model for the whole Occitanie region, the age- and sex-standardized PAH rate was positively associated with premature mortality, with the proportion of workers, with access to GPs, with the number of specialist encounters by patients, and with the number of nurse encounters in the general population (Table 3).

**Table 3:** Determinants of the Rate of Potentially Avoidable Hospitalizations

	Occitanie Estimate (95%CI)	Zone 1 Estimate (95%CI)	Zone 2 Estimate (95%CI)
Intercept	1.6* (0.5;2.6)	1.1 (− 0.14;2.5)	4.4* (2.6;6.5)
Density of nurses	− 0.03* (− 0.04;-0.02)	−0.018* (− 0.036;-0.001)	−0.05* (− 0.07;-0.03)
Access to GP	0.006* (0.0006;0.01)	0.006. (−0.0005;0.014)	0.011* (0.004;0.017)
At least 1 encounter with GP			−2.9* (− 4.4;-1.3)
Specialist encounters (patients)	0.9* (0.3;1.6)	1.16* (0.17;2.4)	
Nurse encounters (population)	0.5* (0.1;0.9)		2.0* (1.2;2.8)
Workers	0.038* (0.013;0.06)		0.03 (−0.002;0.06)
CMUc recipients	0.036 (−0.008;0.08)	0.036 (0.001;0.07)	
Unemployment	−0.1* (− 0.17;-0.04)		
Premature mortality	0.005* (0.002;0.008)	0.005* (0.002;0.008)	0.004* (0.0002;0.007)
Rho	0.24* (0.12;0.36)	0.21* (0.04;0.35)	0.16 (−0.02;0.32)
Moran test for residual autocorrelation (p-value)	0.4	0.063	0.51
Breusch-Pagan test for heteroscedasticity (*p*-value)	< 0.001	0.082	0.35

Data shown are parameter estimates and 95% confidence intervals (2.5 and 97.5% quantiles obtained from 5000 MCMC simulations)

*indicates a p-value < 0.05

Rho is the endogenous interaction effect, the spatial autoregressive parameter indicating the intensity of the interaction between neighboring PAH rate observations

The age- and sex-standardized PAH rate was negatively associated with the unemployment rate and with the density of nurses. Regarding the latter, a one unit increase in the density of nurses was associated with a decrease of 0.029 units in the PAH rate (*p* < 0.05). However, the Breusch-Pagan test rejected the null hypothesis of homoscedasticity, indicating significant conditional heteroscedasticity. For this reason, and because we a priori knew that the Occitanie region was very heterogeneous in terms of socio-economic status, we decided to split the region into 2 subregions and to run the model again.

In Zone 1, the age- and sex-standardized PAH rate was positively associated with premature mortality and with the number of specialist encounters by patients. It was negatively associated with the density of nurses. Regarding the latter, a one unit increase in the density of nurses was associated with a decrease of 0.018 units in the PAH rate (*p* < 0.05). Lastly, in Zone 2, the age- and sex-standardized PAH rate was positively associated with premature mortality, with access to GPs, and with the number of nurse encounters by patients. It was negatively associated with the proportion of the population having at least one GP encounter and with the density of nurses. Regarding the latter, a one unit increase in the density of nurses was associated with a decrease of 0.049 units in the PAH rate (p < 0.05). In both Zone 1 and Zone 2, the Breusch-Pagan test did not reject the null hypothesis of homoscedasticity and there was no remaining spatial autocorrelation.

The other variables related to primary care were not significantly associated with the PAH rate.

## Discussion

### Main results

In this regional study based on 2014 hospital discharge data and ambulatory care claims data, the rate of PAH amounted to 4.85 admissions per year per 1000 population with considerable geographic variation. This result is similar to a recent national report using the same definition [29] and was lower than previous research evidence based on a broader definition of PAH [12].

This study suggests that the underlying mechanisms leading to PAH depend on socio-economic characteristics in region Occitanie. To account for this spatial heterogeneity, we split the region into two sub-regions homogeneous in terms of socio-economic traits and fitted a spatial-lag model for each zone. (The Breusch-Pagan test for heteroscedasticity discarded the spatial lag model for the entire region Occitanie, whereas it showed no significant heteroscedasticity in the models fitted in the two zones.)

The determinants common to both zones were the density of ambulatory care nurses, negatively correlated to PAH, and the premature mortality rate, a proxy of population health status, positively correlated to PAH.

The other determinants differed between the two models.

In zone 1, the more socio-economically deprived zone, the number of specialist encounters was positively correlated to PAH rate. In zone 2, the socio-economically prosperous zone, number of nurse encounters and access to GP were positively correlated to PAH rate.

In both zones, there was a significant and negative association between the density of ambulatory care nurses and the standardized PAH rate. Although the international literature about the impact of ambulatory care nurses on the management of patients with chronic conditions is mixed [30], this is consistent with previous research in France [12] and abroad [31], suggesting that case management by ambulatory care nurses could result in reduced PAH rate. Patients with multiple chronic conditions such as chronic heart failure or COPD and living in low-density areas could be at higher risk of complications or could be more frequently seeking inpatient care.

In Zone 1 and 2, the premature mortality rate was significantly and positively associated with the standardized PAH rate. These results are in line with previous work showing that patients with multiple chronic conditions experience a higher PAH risk [5, 12]. The proportion of the population exempted for at least one severe chronic condition was not associated with the PAH risk in our study. However, this variable was strongly linked to the premature mortality rate.

The increased PAH risk in geographic areas with a poor socio-economic status is well known [5, 9, 19, 32]. Here, this effect was shown only in the Occitanie region model, which was expected because we reduced the socio-economic variation by splitting the region into two homogeneous zones in terms of socio-economic variables.

Regarding primary care, the set of variables associated with the PAH risk differed significantly between Zone 1 and 2. In the former, the number of encounters with a specialist physician for patients with at least one encounter was significantly and positively associated with PAH risk. Even though the evidence regarding the link between specialist care and PAH is unclear [15], the variable could be interpreted as a proxy for the intensity of specialist care. Hence, it could be an indicator of disease severity instead of access to specialist care. In Zone 2, PAH risk was positively associated with access to GPs and with the number of encounters with a nurse in the general population. In addition, PAH risk was negatively associated with the proportion of the population having at least one GP encounter per year. This complex pattern might be a result of the entangled effects of intensity of care due to disease severity (i.e., nurse encounters in the population and access to GPs) and of the protective effect of actual utilization of GP care (i.e., proportion of the population with at least one GP encounter). Indeed, published evidence suggests that greater utilization of primary care could reduce the PAH risk [1, 15]. Another possible interpretation of this result is that, in a socio-economically favored areas, patients are more prone to visit their GP in a timely manner, so that GP visits would have an effective preventive action. These results might also be explained by variation in the respective roles of GPs and ambulatory care nurses in the management of patients with chronic diseases in France.

It is noteworthy that the spatial lag model fitted in the Occitanie region, which was discarded due to remaining heteroscedasticity, was more or less a combination of the significant variables of the models in zone 1 and 2.

### Strengths and limitations

When analyzing PAH determinants, one important issue is the definition used [33]. In this study, we used the AHRQ definition because (i) it has been adapted to the ICD 10 classification [4], (ii) it distinguishes between acute and chronic conditions and (iii) the French quality indicator recently published is a direct adaptation from the AHRQ indicator [29]. Another common concern is the geographically structured nature of the data that requires specific modeling methods to obtain unbiased and robust estimates [23, 34, 35]. We used a spatial-lag model to account for this. Finally, we included a wide range of primary care-related variables, including densities, access indices, and utilization indices derived from individual-level utilization data. Considering the complexity of the underlying causal phenomenon [15], this approach prevented us, at least partly, from introducing potential confounding.

However, this study suffers from several limitations. First, hospital discharge data is prone to misclassification bias when diagnoses are not properly coded. Nevertheless, the French hospital discharge database is considered reliable, especially since 2007 [36]. Second, the results showed an unexpected positive association between the number of nurse and specialist encounters and PAH risk. The most straightforward interpretation would be that patients in poorer health status use more primary care, and this suggests that the adjustment on health status might be imperfect [15]. However, this point does not threaten the overall validity of the results. Last, we were not able to adjust for factors that could contribute to the understanding of PAH but are unavailable in France: integration and continuity of care [11, 13], telemedicine programs [37, 38] and individual care seeking patterns. A possible way to address these limitations in future work would be to rely on clinical and patient-reported data in addition to claims data.

### Policy implications

PAHs are typical high-cost and avoidable episodes of care and threaten the equity, quality and efficiency of health care systems. As such, they are on the policy agendas of several countries [4]. In France, PAHs are now routinely monitored as part of the new set of quality indicators [29] and hence disentangling the multiple determinants of PAH is of high interest to policy makers at the national and regional levels. Our results confirm that increasing the density of nurses in selected geographic areas could contribute to a reduction in the PAH rate. For the first time in France, our results suggest a protective role on the proportion of the general population having actually seen a GP. This effect had not been seen in previous studies [12, 19]. From a policy perspective, a joint intervention combining ambulatory care nurses and GP utilization among patients with chronic conditions might have a stronger effect on PAH rates than an intervention limited to the density of nurses.

Another important implication is that when it comes to modeling PAH risk, the way geographic areas are built matters. In spite of the implementation of spatial-lag models, we chose to split the region into two zones, primarily because these two areas differ so much in terms of socio-economic models. If we had not done so, the protective effect of GP utilization would not have been observed in Zone 2. Another way to tackle this issue would have involved introducing group wise heteroscedasticity into the Occitanie region model, but we felt that the explicit separation into two geographical areas would be helpful for policy makers. Indeed, this allowed for the identification of specific local determinants of PAH and argues for a local implementation of public health policies. It also sheds light on the importance of the choice of the method to build geographic areas [39].

## Conclusions

In this study, at the ZIP code level in France PAH risk was explained by socio-economic determinants, the health status of the population and several primary-care related variables. Regarding the latter, the protective role of GP encounters was shown only in the socio-economically favored zone. This finding highlights the importance of tackling spatial heterogeneity when assessing the role of primary care on PAH.

## Supplementary information

**Additional file 1.** Table that shows the definition of potentially avoidable hospitalizations.

**Additional file 2.** Table that presents all the variables considered in the statistical analysis.

## Data Availability

The use of PMSI and SNIIRAM databases was approved by the National Committee for data protection (Commission Nationale Informatique et Libertes) and we are not allowed to share them. The rest of the data are available from open websites: French Ministry of Health [24], the National Institute for Statistics and Economic Studies [25], and the Regional Health Observatory [26].

## Abbreviations

AHRQ: Agency for healthcare research & quality
APL: Accessibilite Potentielle Localisee
CMUc: Couverture Maladie Universelle
COPD: Chronic obstructive pulmonary disease
GP: General practitioner
OLS: Ordinary least square
PAH: Potentially avoidable hospitalizations
PMSI: Programme de Médicalisation des Systèmes d’Information
SNIIRAM: Systeme National d’Information Inter-Regimes de l’Assurance Maladie
