# Supplementary material for: The effect of primary care on potentially avoidable hospitalizations in France: a cross-sectional study
Source: BMC Health Serv Res. 2020 Mar 31;20:268. doi: 10.1186/s12913-020-05132-6 (PMC7106616; doi:10.1186/s12913-020-05132-6)
Supplement: Supplementary file 1 — Additional file 1. Table that shows the definition of potentially avoidable hospitalizations. [file 12913_2020_5132_MOESM1_ESM.pdf]

Supplemental Digital Content 1: Definition of Potentially Avoidable Hospitalizations (source : ECHO Handbook : http://www.echo-health.eu/handbook/PAH_asthma.html)

	Primary diagnoses		Secondary diagnoses		Procedures	
	Inclusions	Exclusions	Inclusions	Exclusions	Inclusions	Exclusions
**Asthma in Adults**. Aged 18 and older	J45 J46<br><br>OR<br><br>J960 IF "diag2-30"=A		A) J45	Pregnancy, childbirth and the puerperium: O00-099<br><br>CHF: I50 I0981 I110 I130 I132<br><br>Cystic fibrosis: E840-E849 Q251-Q254 Q30 Q31 Q32 Q33 Q34 Q39 Q893 P26<br><br>Mental disorders: F10-F19 F20 F21 F22 F23 F24 F25 F29 F30 F31 F32 F33 F34 F38 F39 F40-F45 F44 F48 F50-F52 F54 F60 F63 F68 F28 F53 F55 F59 F61 F62 F69 F28 F53 F55 F59 F61 F62 F69<br><br>Respiratory diseases: J47 J8410 J98 J99 COPD: J411 J418 J42 J43 J44 J47		
**Congestive Heart Failure (CHF).** Aged 40 and older	I09.9 I11.0 I13.0 I13.2 I50			Pregnancy, childbirth and the puerperium : O00-099<br><br>COPD: J411 J418 J42 J43 J44 J47<br><br>Ischaemic disease: I20 I21 I22 I24.0 I24.8<br><br>Kidney failure: I12 I13.1 N17 N18 N19		

**Chronic obstructive pulmonary disease** (COPD). Aged 18 and older	J42 J43 J44 J47 J411 J418<br><br>OR<br><br>J20 IF DX= "A)" or J40 IF DX= "A)" J960 IF DX= "B)" or<br><br>J969 IF DX= "B)"		A) J42 J43 J44 J47 J411 J418<br><br>B) J42 J449 J47	Pregnancy, childbirth and the puerperium: O00-099<br><br>CHF: I50 I0981 I110 I130 I132<br><br>Cystic fibrosis: E840-E849 Q251-Q254 Q30 Q31 Q32 Q33 Q33 Q34 Q33 Q34 Q34 Q34 Q39 Q893 P26<br><br>Mental disorders: F10-F19 F20 F21 F22 F23 F24 F25 F29 F30 F31 F32 F33 F34 F38 F39 F40-F45 F44 F48 F50-F52 F54 F60 F63 F68 F28 F53 F55 F59 F61 F62 F69		
**Dehydration in elderly people (DH).** Aged 65 and older	E86 E870 E871					
**Diabetes short-term complication.** Aged 40 and older	E10.0 E10.1 E11.0 E11.1 E13.0 E13.1			Pregnancy, childbirth and the puerperium: O00-099<br><br>Mental Disorders: F10-F19 F20 F21 F22 F23 F24 F25 F29 F30 F31 F32 F33 F34 F38 F39 F40-F45 F44 F48 F50-F52 F54 F60 F63 F68 F28 F53 F55 F59 F61 F62 F69		
**Angina without procedure.** Aged 40 and older. Urgent admissions	I20.0 I24.0 I24.8 I20.1 I20.8 I20.9			Pregnancy, childbirth and the puerperium: O00-099		Cardiac Procedures
