# Supplementary material for: The effect of primary care on potentially avoidable hospitalizations in France: a cross-sectional study
Source: BMC Health Serv Res. 2020 Mar 31;20:268. doi: 10.1186/s12913-020-05132-6 (PMC7106616; doi:10.1186/s12913-020-05132-6)
Supplement: Supplementary file 2 — Additional file 2. Table that presents all the variables considered in the statistical analysis. [file 12913_2020_5132_MOESM2_ESM.pdf]

SDC 2: Presentation of all the variables considered in the statistical analysis

	Year	Source	Standardisation	Selected by PCA	Significant in at least one model
**Variable analysis**					
Number of potentially avoidable hospitalizations per 1000 persons	2014	PMSI	Age and sex direct standardisation		N/A
**Socio-economic and demographic data**					
Deprivation index (Fdep09)	2009	Inserm	no	Yes	
Population structure (age and sex)	2012	Insee	no		N/A
Median pre-tax household income	2012	Insee	no	Yes	
Proportion of taxable households	2012	Insee	no		
Proportion of single-parent families	2012	Insee	no	Yes	
Education level (% of population with baccalaureate)	2012	Insee	no	Yes	
Distribution of the population by occupational groups	2012	Insee	no	Yes	Yes
Unemployment rate	2012	Insee	age and sex direct standardisation	Yes	Yes
Proportion of main residences with bathroom, bath or shower	2012	Insee	no		
Proporion of population living in isolated rural areas	2012	Insee	no	Yes	
Proportion of CMU-c reciptients	2016	Health Insurance	no	Yes	Yes
**Proxy of health status**					
Mortality rate	2008-2012	ORS	age and sex direct standardisation		
Premature mortality rate	2008-2012	ORS	age and sex direct standardisation	Yes	Yes
Exemption for chronic disease	2013	ORS	no	Yes	
**Primary care supply**					
Density of general pratictioners	2015	ARS	no	Yes	

	Year	Source	Standardisation	Selected by PCA	Significant in at least one model
Density of ambulatory care nurses	2015	ARS	no	Yes	Yes
Density of physiotherapists	2015	ARS	no		
Density of dentists	2015	ARS	no		
Density of cardiologists	2015	ARS	no		
Density of endocrinologists	2015	ARS	no		
Density of ophtalmologists	2015	ARS	no		
Density of pulmonologists	2015	ARS	no		
Access to general pratictioners	2010	DREES	no	Yes	Yes
Access to ambulatory care nurses	2010	DREES	no	Yes	
Access to physiotherapist	2010	DREES	no	Yes	
Access to dentist	2010	DREES	no		
Access to ambulotary care specialist physicians : Gynecologist, ophthalmologist, pediatrician, pharmacy, Midwife, Psychiatrist	2010	DREES	no		
Travel time to emergency department	2013	ARS	no	Yes	
Travel time to acute hospital	2013	ARS	no		
Travel time to medical group practice	2013	ARS	no	Yes	

	Year	Source	Standardisation	Selected by PCA	Significant in at least one model
Ambulatory care center	2013	ARS	no		
Nursing home service	2013	ARS	no		
**Primary care utilization**					
**3 indicators :**					
Rate of population with at least one encounter in the year	2013	Health Insurance (SNIIRAM)	age and sex indirect standardisation (index)		
Number of encouters per person (general population)	2013	Health Insurance (SNIIRAM)	age and sex indirect standardisation (index)		
Number of encounters in the year (patients)	2013	Health Insurance (SNIIRAM)	age and sex indirect standardisation (index)		
**Calculated for :**					
General praticitioners	2013	Health Insurance (SNIIRAM)		Yes	Yes - Rate of encounters
Ambulatory care nurses	2013	Health Insurance (SNIIRAM)		Yes	Yes - Encouters (population)
Physiotherapists	2013	Health Insurance (SNIIRAM)		Yes	
Dentists	2013	Health Insurance (SNIIRAM)		Yes	
Specialist physicians: cardiologists, pulmonologists, endocrinogists, nephrologists	2013	Health Insurance (SNIIRAM)		Yes	Yes - Encounters (patients)

Source's list

DREES - Ministry of health

INSEE - National Institute for Statistics and Economic Studies

ORS - Regional health observatory

Health Insurance (National health insurance information system - SNIIRAM)

ARS - Regional Health Agency of Occitanie

Inserm - https://public.opendatasoft.com/explore/dataset/metropole-indice-de-defavorisation-sociale-fdep-a-lechelle-de-liris-2009-inserm/?flg=fr

PMSI - Programme de Médicalisation des Systèmes d'information - National hospital discharge database
